# A report of a family with an MSH2 mutation and prostate cancer

**DOI:** 10.1186/1897-4287-9-S1-P29

**Published:** 2011-03-10

**Authors:** Erin A Mundt, Sara C Knapke, Robert Hopkin

**Affiliations:** 1 The Hereditary Cancer Program, Division of Human Genetics, Cincinnati Children’s Hospital Medical Center, Cincinnati, OH 45229, USA

## Background

There have been conflicting reports regarding the possibility of an association between Lynch syndrome and prostate cancer. Some reports indicate that individuals with mismatch repair gene mutations may be at an increased risk of prostate cancer, while others show little to no evidence of an association. We are reporting a family with multiple cancers including colorectal, endometrial, ovarian, brain, skin and prostate.

## Case report

The proband initially presented with a history colorectal cancer at the age of 39. He also had a history of multiple sebaceous adenomas and squamous cell carcinomas (keratocanthoma type). Initial testing included germline mutation analysis of the MH1, MSH2, and MSH6 genes as tumor sample for MSI and IHC was not available. Genetic test results revealed the IVS5+3A>T mutation in MSH2. Based on the family history, including multiple aggressive skin cancers, this mutation is consistent with a diagnosis of Muir-Torre syndrome. As expected, the MSH2 mutation tracks with colorectal, endometrial and ovarian cancers in the family. Interestingly, the MSH2 mutation appears to be tracking with prostate cancer in the family as well. Numerous men in this family have been diagnosed with aggressive, early onset prostate cancer. The proband’s father, an obligate carrier of the MSH2 mutation, was diagnosed with prostate cancer at the age of 45 and passed away at the age of 49. A paternal uncle was diagnosed with prostate cancer at the age of 41 and passed away of a brain tumor at the age of 60. He passed away before having genetic testing so it is not clear that he carried the familial MSH2 mutation; however, the history of the brain tumor is suspicious. Two additional uncles were diagnosed with prostate cancer at later ages (55 and 65). The uncle diagnosed at age 55 has a daughter that was diagnosed with endometrial cancer in her 30s. The proband also reports three first cousins with prostate cancer. One was diagnosed in his 40s and the other two in their 50s. One of the cousins diagnosed with prostate cancer in his 50s was also diagnosed with colorectal cancer at the age of 42 and does carry the familial MSH2 mutation.

## Conclusion

In this family a mutation in the MSH2 mismatch repair gene appears to be associated with an increased risk for prostate cancer, indicating that additional research may support the association between prostate cancer and Lynch syndrome.

